# 

**Published:** 1902-01-15

**Authors:** 


### SUBJECT INDEX TO VOLUME LVI.

ADDRESSES AND PAPERS.

Address of President, C. R. Rowley, 271.
Address of President, C. H. Oakman, 325.
Address of President, H.F. Harvey, 25.
A Little of Several Things, C. H. Warboys, 294.
A Study in Bleaching Teeth, N. S. Hoff, 452.
Cohesion of Gold, C. A. Hawley, 79.
Dentistry as a Specialty of Medicine, O. N. Heise, 183.
Dental Education in the Public Schools, W. L. Mercer, 300.
Dental Diseases an Etiological Factor in Affection of the Eye, Cundell Juler, M.D., 23.
Dental Instruments, J. A. Watling, 607.
Diagnosis in Dental Practice, J. Taft, 8.
Essential Oils, D. Stern, 446.
Extracting from an Orthodontist's Point of View, 1.
First Molar Necessary to Dentist's Financial Success, C. M. Wright, 247.
Hæmophilia, S. D. Ruggles, 27.
How to Construct an Upper Vulcanite Denture Quickly, C. H. Warboys, 571.
Inportance of Conserving the First Molars, A. C. Runyan, 560.
Irregularities and How to Correct Some of Them, J. H. Stafflett, 553.
Making an Inlay Matrix, H. J. Bosart, 95.
Mechanical Device for Cleft Palate, H. J. Jaulusz, 565.
Medical Specialties, E. I. Backus, 283.
Mistakes and Failures in Crown and Bridge-work, F. E. Logan, 163.
Moral Side of Dentistry, (third paper), G. W. Clapp, 55.
Nitrous Oxid Gas, S. Straith, 541.
Nitrous Oxid and Oxygen, D. H. Ziegler, 66.
Oral Antisepsis in Relation to Preventive Medicine, C. M. Wright, 39.
Past and Future of Dentistry, F. H. Essig, 280.
Porcelain, its Possibilities as a Filling Material, W. T. Reeves 379.
Preventive Dentistry, C. M. Wright, 595.
Prosthetic Dentistry, A. G. Rose, 196.
Pyorrhea Alveolaris, E. B. Newell, 331.
Real Attitude of the Dental Specialty toward Medicine, F. W. Sage, 236.
Recurrence of Caries under Gold Fillings, H. A. Smith, 229.
Reminiscences and Things, J. S. Cassidy, 354.
Some Dental History, A. T. Metcalf, 487.
Some Things the General Practitioner ought to take into Account in Orthodontia, C. B. Blackmarr, 599.
Some Things that Make a Dentist Professional, N. S. Hoff, 217.
Suppurative Diseases of the Maxillary Sinus, H. M. King, M.D. 403.
The Ladies, A. L. LeGros, 308.
The X-Ray as Applied to Dental Practice, L. E. Custer, 433.
The X-Ray in Dentistry, P. M. Hickey, M.D., 421.
What Art has Done for Dentistry, etc., B. J. Cigrand, 569.
Will a High Preliminary Requirement Remove Existing Evils? G. S. Junkerman, 109.

### REPRINTS.

Adrenalin as an Antidote for Cocain Solutions, C. A. Elsberg, M. D., 576.
Antiseptic Mouth Wash, 256.
Bleaching Teeth with Hydrogen Dioxid, Joseph Head, 248.
British Dental Association compared with the National Dental Association, W. H. Trueman, 578.
Calcium Chlorid to Prevent Hemorrhage, 583.
Can the Pulp be Anesthetized by Injecting the Peridental Structures? T. W. Brophy, 518.
Care of the Deciduous Teeth, 145.
Cocain in Dentistry, E. R. Carpenter, 515.
Crawford Crown, 149.
Dental Education, Joseph Griffiths, M.D., 117.
Effect of Vulcanite on Certain Metals, 203.
Entrance Requirements to German Dental Schools, 585.
Extracting First Molars, H. A. Pullen, 522.
Filling a Mesio-occlusal Cavity, E. K. Wedelstaedt, 131.
Gutta Percha, its Value in Dentistry, 311.
High and Low-fusing Porcelains, 611.
Impressions and Bites, W. H. Stowe, 147.
Is it Worth While to Try to Save Teeth? S. A. Hopkins, 143.
Judge Scores a Dental School, 582.
Obtunding Dentin, 206.
Ohio Dental Law, 360.
Orthodontia, E. H. Angle, 528.
Plastic Fillings of the Metallic Salts, E. Schmitt, 584.
Porcelain Fillings, W. C. Capon, 96.
Pyorrhea Alveolaris, R. Good, 254.
School Children's Teeth Examination, 151.
Soldering Hint, 359.
Solidified Formaldehyde, F. B. Lawrence, 498.
Somnoform, 573.
Statistical Study of Local Anesthesia, M. I. Schamberg, 137.
The Matrix Retainer in Plastic Fillings, 252.
The Young Dentist has Come, 42.
Work of Dental Corps at Fort Presidio, J. S. Marshall, 530.

### EDITORIAL.

Amendment of Ohio Dental Law, 366.
Arsenical Poisoning, 156.
Bill for Dental Surgeon in the Navy, 102.
Careless Writing, 533.
Central Jersey Association's Novel Meeting, 587.
Cocain, 533.
College Commencements, 258.
Conduct of Dental Societies, 429.
Corrections, 207, 630.
Dental News, 156.
Fund for Dental Scientific Research, 313.
Graduates of Dental Colleges, 366.
Harlan's Loving Cup, 208.
Harlan, Dr. A. W., retires from the Editorial Management of the Dental Review, 102.
Institute of Dental Pedagogics, 101.
International Dental Federation, 209.
Mat Gold, 508.
Navy Bill, 629.
Ohio New Dental Law, 314, 366.
Ohio Dental Journal burned out and changes name, 103.
Pink Teeth, 157, 260.
Proceedings of National Dental Association, 258.
Reduction in Subscription Price of Dental Journals, 586.
Students' Hall of Washington University at St. Louis, 587.
St. Luke's Hospital, 257.
St. Luke's Hospital and the British Dental Journal, 155.
The National Conventions, 472.
The New York M.D.S. Degree, 534.

BIBLIOGRAPHY

Abbott's Bacteriology, 369.
American Dental Journal, 539.
Broomell's Anatomy, 587.
Care of the Teeth, 476.
Medical Book News, 539.
Porcelain Teeth, 538.
Principles and Practice of Operative Dentistry, J. S. Marshall, 48.
Questions and Answers, F. J. S. Gorgas, 47.

ANNOUNCEMENTS.

American Dental Society of Europe, 262.
American Society of Orthodontists, 210, 475.
Central Michigan Dental Association, 265, 476.
Chicago Odontographic Society, 104.
Chicago Dental Society, 266.
Grand Rapids Dental Society, 158.
Indiana State Society, 315.
Illinois State Society, 159, 209.
Illinois State Society Officers, 314.
Institute of Dental Pedagogics, 631.
Kentucky State Society Officers, 368.
Michigan State Society, 265, 368.
Missouri State Society, 158.
National Association of Dental Faculties, 315.
National Dental Association, 264, 315.
National Dental Association, Southern Branch, 474.
New Jersey State Society, 264.
Northern Ohio Society, 210.
Ohio Board of Examiners, 589.
Ohio State Society Officera Elected, 105.
Ohio Valley Dental Society, 158.
Ohio State Society, 588.
Pennsylvania Dental Examiners, 264, 589.
South Carolina State Society, 210.
Southwestern Michigan Dental Society, 158, 265, 476.
West Virginia Dental Board of Examiners, 266.

OBITUARY.

A. M. Callahan, 538.
A. V. Elliot 535.
Charles J. Essig, 104.
J. A. Harris, 632.
H. B. Noble, 259.
A. W. Smith, 537.
J. A. White, 536.

INDENTS.

Abscess Syringe, 370.
Acetylene Gas Explosion, 484.
Acoine, New Anesthetic, 480.
Adrenalin Hypodermically, 323.
Aluminum Linings, 107.
Aluminum Solder, 296.
Amalgam Filling, 591.
Amalgam and Health, 633.
Amalgam Inlay, 374
Amalgam Fillings, Polishing, 477.
Anesthetic Mixture, 431.
Anesthetics, Relative Values, 483.
Annealing Steel, 211.
Anomalies, 378.
Antidote for Formaldehyde, 477.
Antiseptic Cavity Varnish, 211.
Argentamins in Pyorrhea Alveolaris, 268.
Arseneous Acid Antidote, 373.
Arsenical Paste, colored, 316.
Articulation and Occlusion, 51.
Articulating Gold Crowns, 431.
Babbitt-metal, 370.
Backing Porcelain Facings, 431.
Bandless Incisor Crown, 377.
Bleaching Pink Rubber, 633.
Born With Teeth.
Boro-chloretone, 106.
Burns, 371.
Calcium Dioxid, 371.
Calcium Chlorid for Hemorrhage, 430.
Calendula, 1(6.
Calendula for Irritated Gums, 540.
Canal Treatment, 371.
Caroid as a Pulp Digestor, 590.
Carbolic Acid Gangrene, 593.
Capsule Implantation, 375.
Castor Oil for Neuralgia, 160.

Cause of Toxic Effects of Cocain, 317.
Cavity Cutter for Porcelain Teeth, 372.
Cellulitis from Alveolar Abscess, 540.
Chinosal, 52.
Chloroform Death, 317.
Chloroform for Children, 213.
Chloretone to Preserve Cocain Solutions, 267.
Chloretone as a General Anesthetic, 317.
Chromic Acid for Ulcerated Stomatitis, 267.
Clarify Wax, 106.
Cleanliness, 430.
Cleaning Leeth, 591.
Closing Flasks, 53.
Cloudy Mirrors Prevented, 430.
Cooain, Stable Solutions, 321.
Cocain, 262.
Cocain Antidote, 481.
Coffee as a Cause of Pyorrhea Alveolaris, 374.
Collections Possible, 321.
Color of Inlays, 52.
Competition and Ethics, 477.
Contouring Amalgam Fillings with Baby Ribbon, 160.
Crowning a Fractured Root, 215.
Dentist and Public, 481.
Devitalizing Pulp Remnants, 371.
Diagnosing Caries, 370.
Difficult Impressions, 432.
Defining the Extent of Caries, 372,
Disking Enamel Margins, 479.
Drills for Opening Cavities, 52.
Dryness of Cavity Essential when Applying Arsenic, 51.
Dry Investment. 466.
Dry Tooth Brush, 373.
Effect of Gum Chewing on the Teeth, 214.
Electricity as an Anesthetic, 637.
Engine Burs Sharpened, 478.
Filling and Firing Porcelain Inlays, 212.
Filtered Water Dangerous, 485.
Fire Cracking of Porcelain Crowns, 161.
Formaldehyde Antidote, 316.
Fusible Alloy, 318.
Gasoline Burners, 467.
German Dentists against Americans, 480.
Gold Chlorid, 371.
Gold Plating Outfit, 376.
Guaiacol in Capping Pulps. 268.
Guaiacol a Local Anesthetic, 540.
Gutta Percha Separation, 213.
Hands, Care of, 373
Hardening Plaster Casts, 161, 477.
Hare Lip, 636.
Hemorrhage, To Check, 374.
Hemostatics, 316.
Hydrogen Dioxid, To Preserve, 160, 370.
Hydrogen Dioxid, Dangerous, 320.
Hydrogen Dioxid, its Misuse, 590.
Hypodermic Needles, To Clean, 477.
Immediate Root Filling, 635.
Implanting Artificial Roots, 636.
Inflammation at Apex after Devitalizing, 486.
Injecting a Pulp, 479.
Inlay Impressions, 376, 481.
Instrument Sharpener, 466.
International Tooth Crown Co. Suit, 53.
Javanevse Method of Narcosis, 592
Joining the Dental Society, 634.
Limitatians in Cavity Preparation, 319.
Lysol for Sterilizing Instruments, 108.
Malarial Periostitis, 540.
Mallets, 484.
Mat Gold, 479.
Matrix Retainer, 482.
Menstrual Neuralgia, 635.
Menthol Preliminary to Anesthesia, 636.
Mentho-Phenol, 318.
Metal Crowns, 467.
Mouth Wash, 372.
Nargol in Empyemia, 162.
Nausea from Vulcanite Plate, 370.
New Alloy, 50.
New Cure for Pyorrhea, 638.
Obtunding Dentin, 480.
Obviating the Pain of Hypodermic Puncture, 211.
Oil of Cinnamon and Thymol as Antiseptics, 51.
Orthoform for Exposed Pulps, 211

Oxygen with Anesthetics for Old People, 267,
Oxyphosphate of Zinc, Non-corrosive to Pulp, 478.
Oxyphosphate of Zinc, Non-irritant, 371.
Oxysulphate of Zinc Cement, 213,
Painless Removal of Tooth Enamel, 50.
Periostitis, 316.
Peronin, a Dental Anesthetic, 322.
Phenol and Cocain as an Obtundent, 316.
Plaster Impressions Facilitated, 319.
Platinum Solder, 160.
Porcelain Inlays, 485.
Polishing Crowns, 317.
Polishing Regulating Bands, 317.
Preservation of the Third Molars, 161.
Preventing Regulating Appliances from Cutting the Mouth. 372.
Professional Diplomacy, 482.
Pulp Mummification, 50.
Pyorrhea, its Treatment, 432.
Rapid Production of Regulating Plates, 215.
Receding Gums, 633.
Rehardening Steel, 634.
Removing a Pin from the Root, 635.
Removing Plaster from the Hands, 633.
Recovering Swallowed Substances, 430.
Removing Gloss from Porcelain Teeth, 211.
Removing Matrix from Inlay, 540.
Repairing Vulcanite Plate, 466.
Resin as a Protector of Steel, 540.
Root Filling Material, 318.
Rubber Dam on Badly Decayed Teeth, 320.
Rules for Cocain Administration, 269.
Separating Fluid, 482.
Separating Teeth, 50.
Separating and Treating, 478.
Spongy Gums, 634.
Spittoons Cleaned, 634.
Severing Nerves in Trephining the Antrum, 107.
Staining Porcelain Teeth, 160.
Sterilizing Caries, 212.
Stopping Decay at Gum Line, 106.
Sunday Dentistry Prohibited, 480.
Swaging Device, 468.
Tempering, 370.
Temporary Stopping, 430.
Tin Foil on Vulcanite, 634.
Toothache Drops, 633.
Training Children to Care for Their Teeth, 318.
Traumatic Wounds and their Painless Antiseptic Treatment, 323.
Why Amalgam Fillings Change Form, 372.
Vacuum Chambers, 319.
Varnish for Plaster Models, 212.
Varnishes, To Introduce, 478.
Vulcanite Bridge, 54.
X-Ray, 469.
Zinc, To Pour, 375.

AUTOGRAPHIC.

Achillis, Dr., 466.
Ambler, H. L., 78, 84.
Ames, W. V-B., 214, 371.
Angle, E. H., 528.
Armand, M., 267.
Armstrong, P. A., 293.
Arndt, L. 633.
Arnold, Otto, 33, 90,
Atwater, H. G., 146.
Backus, E. I., 283, 293.
Barber, L. L., 115.
Bannister, B., 466.
Barnes, H., 89.
Barrelier, D., 430.
Barrenechea, J. V., 432.
Barton, E. R., 106.
Beach, J. R., 431.
Beauman, J. B., 87.
Bentley, C. E., 621.
Benvenute, Dr. 322.
Black, G. V. 316.
Blake, M. E., 373.
Blackmarr, C. B., 599, 605, 606.
Bogue, E. A., 50.
Bousas, C., 160.
Bosart, H. J., 90, 95, 115.
Brewster, R., 615, 627.
Bradley, J. H., 107,
Bridges, J. S , 613.
Briggs, W. A. 637.
Brophy, R. C., 467.
Brophy, T. W., 123, 411, 417, 518.

Brown, G. M., 467.
Browne, Sir J. C. 130.
Brunton, G., 216.
Bryant Geo., 540.
Buckley, J. P., 461.
Butler, C. R., 19, 88, 93.
Caldwell, Wm., 317.
Callanan, J. R , 192, 234.
Capon, W. C., 96.
Carpenter, Geo. T., 370.
Carpenter, E. R., 515.
Caspar, Dr., 373.
Cassidy, J. S , 74, 234, 243, 354, 450, 451, 459.
Choquet, J., 212.
Chupein, Dr., 375.
Cigrand, B. J., 563, 569, 602, 605.
Clapp, Geo. W., 55.
Cook, J. J., 299, 307, 445.
Cone, W. R., 510.
Crouse, J. N., 245, 482.
Custer, L. E., 113, 433.
Day, C. F., 394, 402.
Dorrance, W. H., 175, 397, 399.
Elliot, W. S. G., 478.
Elsberg, C. A., M.D., 576.
Essig, F. H., 280, 306, 350.
Esterly, C. E., 53.
Fernandez, E. M. S., 213.
Ferguson, W. C., 305.
Fingen, Dr., 638.
Fletcher, M. H., 244.
Foster, Sir M., 129.
Francis, E. A., 213.
Girwood, J., 215.
Gallie, Don M., 621.
Grant, W. E., 319.
Griffith, Jos , M.D., 117.
Griffis, Dr., 484.
Good, G., 477.
Good, R., 254, 432.
Gorgas, F. J. H., 47.
Goslee, H. J., 51, 623.
Grieves, C. J., 634.
Guilford, S. H., 161.
Hall, L. P., 180.
Hanson, C. P., 550.
Harlan, A. W., 160.
Harnstein, S., 371.
Harvey, H. L., 25.
Haskell, L. P., 370, 634.
Hawley, C. A., 79, 91.
Head, J., 248.
Heidbrink, J. A., 261.
Heise, O. N., 183, 195, 200, 235, 245, 451.
Henderson, Dr., 306.
Hesse, Prof., 125.
Hetrick, F. O., 484, 510, 513.
Hickey, P. M., 421, 443, 469.
Hines, P. F., 350.
Hirschfield, Wm., 479.
Hoff, N. S., 172, 217, 292, 297, 307, 346, 348, 452, 479.
Hofheinz, R. H., 478, 482.
Holland, C. P., 268.
Honey, E. A., 307, 552, 610.
Honey, S. W., 293, 300.
Hood, C. O., 52.
Hooper, N. E., 553.
Houghton, Dr., 267.
Howell R. B., 468.
Howorth, F. A., 52.
Hughes, W. R., 371.
Hunter, F. A., 190, 199.
Hutchinson, R. G., 252.
Igel, H., 318.
Inglehart, T. N., 147.
Inglis, O. E., 320.
Jackson, W. H., 178, 182.
Jaulusz, H. J., 565.
Jenkins, N. S., 316.
Johnson, H. H., 151.
Johnson, J. A., 320, 431.
Johnson, C. N., 527.
Junkerman, G. S., 109, 116.
Juler, Cundell, 23.
Keyes, W. B., 268.
Kimball, D., 479.
King, H. M., M.D., 403, 420.
Kirk, E. C., 126.
Kumler, A. A., 244.
Lanphear, O. E., 306.
Laurence, F. B., 498.
LeCrone, Dr., 53.
LeGros, A. L., 293, 308.
Levin, J., 318.
Loeffler, E. T., 391.
Logan, F. E., 163, 180.
Low, F. W., 372.
Madden, J. J., 372.
Magill, W. F., 108.
Morey, A. E., 611.
Marshall, J. S., 48, 530.
Matteson, A. E., 377.
Mourel, Dr., 317.
McLain, Dr., 483.
Megrow, J. R., 540, 634, 636.

Mercer, W. L., 300.
Metcalf, A. T., 487.
Meyers, I., 466.
Miller, W. D., 108.
Mitchell, Wm., 161.
Molyneaux, G., 200, 201.
Moore, E. C., 396.
Morrison, Dr., 633.
Narichal, Dr., 540.
Newell, E. B., 331, 349, 350.
Noble, S. S., 508.
Noel, L. G., 630.
Noyes, Edmund, 627.
Nyman, J. E., 625.
Oakman, C. H., 325, 348, 353, 418.
Osman, Dr. 52.
Ottolengui, R., 527.
Parker, F. M. 147.
Parker, J., 416.
Payne, R. E. 376.
Perry, S. G., 374, 376.
Pierce, C. N , 527.
Platt, F. L., 146.
Price, W. A., 630.
Pryn, C. P., 591.
Pullen, H. A., 522.
Raymond, E. H., 480 .
Reeves, W. T., 379, 402, 485, 619.
Renz, S. J., 510.
Rix, T. G., 292, 299 306, 345, 348, 551, 557, 610.
Rockey, J. A., 372.
Roe, C. B., 305, 559.
Rose, A. G., 196, 451.
Ruggles, S. D., 20, 27, 37.
Runyan, A. C., 560, 564.
Sage, F. W., 193, 201, 236.
Schmitt, E., 584.
Schultze, Dr., 54.
Smith, D. D., 635.
Smith, H. A., 17, 36, 114, 116, 195, 229, 235, 450, 451.
Smith, H. T., 192.
Soudern, P. F., 431.
Spence, S. J., 160, 161, 211, 478.
Starr, Dr., 371.
Stephan, J. S., 76, 89.
Stern, D., 446, 452.
Straith, S., 351, 353, 541.
Stafflet, J. H., 553, 559.
Swartz, F. G., 157.
Taft, J., 8, 21, 34, 90, 176, 400, 415, 527, 558, 564.
Taylor, W. J., 145.
Tileston, H. B., 319.
Todd, F. C., 323.
Townsend, E. L., 319.
Trueman, W. H., 578.
Tuller, R. B., 635.
Walker, W. W., 638.
Wallace, W. J., 107.
Wallis, Dr., 430.
Warboys, C. H., 294, 300, 306, 352, 563, 571.
Watling, J. A., 559, 607.
Watson, M. T., 1, 399.
Way, T. I., 244.
Waye, E. J., 203.
Webster, A. E., 261.
Wedelstaedr, E. K., 478, 591.
Welch, Dr., 605.
Werner, Dr., 211.
White, S. M., 307, 350, 551.
Whitslar, W. H., 16.
Williams, J. L., 370, 371, 636.
Williams, R. O., 430.
Williams, W. M., 191.
Willmott, J. B. 372.
Wilson, G. H., 77.
Wippern, A. C., 162.
Wise, C. A., 307, 352, 353.
Wood, A. L., 485.
Woohead, Dr. S., 124.
Wright, C. M., 39, 189, 244, 245 247, 595, 634.
Ziegler, D. H., 66, 78.

### ANNOUNCEMENTS.

### SOUTHERN BRANCH NATIONAL DENTAL ASSOCIATION.

The fifth annual meeting of the Southern Branch of the National Dental Association, will be held at Atlanta, February 18, 1902. The Association will be in session four days. Atlanta is now the best located and equipped city in the south for holding such a meeting. This fact assures a large attendance. The South-Eastern Passenger Association will give a rate of one and one-third fare for the round trip. All members are earnestly requested to be present.

C. L. ALEXANDER,
*Cor. Sec'y, S. B. N. D. A.*
Charlotte, N. C.

